# Dual functions of TAF7L in adipocyte differentiation

**DOI:** 10.7554/eLife.00170

**Published:** 2013-01-08

**Authors:** Haiying Zhou, Tommy Kaplan, Yan Li, Ivan Grubisic, Zhengjian Zhang, P Jeremy Wang, Michael B Eisen, Robert Tjian

**Affiliations:** 1 Department of Molecular and Cell Biology, Howard Hughes Medical Institute, University of California, Berkeley, Berkeley, United States; 2 Li Ka Shing Center For Biomedical and Health Sciences, CIRM Center of Excellence, University of California, Berkeley, Berkeley, United States; 3 Department of Molecular and Cell Biology, California Institute of Quantitative Biosciences, University of California, Berkeley, Berkeley, United States; 4 School of Computer Science and Engineering, The Hebrew University of Jerusalem, Jerusalem, Israel; 5 Janelia Farm Research Campus, Howard Hughes Medical Institute, Ashburn, United States; 6 UC Berkeley-UCSF Graduate Program in Bioengineering, Department of Molecular and Cell Biology, California Institute of Quantitative Biosciences, University of California, Berkeley, Berkeley, United States; 7 Department of Animal Biology, University of Pennsylvania School of Veterinary Medicine, Philadelphia, United States; University of California-San Diego, United States

**Keywords:** ChIP-seq, RNA-seq, adipogenesis, C3H10T½, TAF7L, differentiation, Mouse

## Abstract

The diverse transcriptional mechanisms governing cellular differentiation and development of mammalian tissue remains poorly understood. Here we report that TAF7L, a paralogue of TFIID subunit TAF7, is enriched in adipocytes and white fat tissue (WAT) in mouse. Depletion of TAF7L reduced adipocyte-specific gene expression, compromised adipocyte differentiation, and WAT development as well. Ectopic expression of TAF7L in myoblasts reprograms these muscle precursors into adipocytes upon induction. Genome-wide mRNA-seq expression profiling and ChIP-seq binding studies confirmed that TAF7L is required for activating adipocyte-specific genes via a dual mechanism wherein it interacts with PPARγ at enhancers and TBP/Pol II at core promoters. In vitro binding studies confirmed that TAF7L forms complexes with both TBP and PPARγ. These findings suggest that TAF7L plays an integral role in adipocyte gene expression by targeting enhancers as a cofactor for PPARγ and promoters as a component of the core transcriptional machinery.

**DOI:** http://dx.doi.org/10.7554/eLife.00170.001

## eLife digest

The development of a single fertilized egg into a highly complex animal is determined by its genome, with a process called differential gene regulation exerting exquisite control over gene expression to ensure that various specialized cells are generated and that many types of tissue are produced. However, the mechanisms responsible for controlling gene expression and, therefore mammalian development, are poorly understood.

Researchers have developed a number of in vitro cell culture models to elucidate the details of differential gene regulation, and this approach has been used to characterize adipocytes—cells that store energy in the form of fat—for close to two decades. The formation of adipocytes, a process known as adipogenesis, has been extensively studied, but there remain major gaps in our knowledge: for example, the identities of many of the transcriptional regulators that are responsible for the differentiation of mesenchymal stem cells into adipocytes remain a mystery. This task is complicated by the fact that some of these regulators are involved in the differentiation of multiple cell lines, and that some of them also have multiple roles in the generation of a single cell type. In addition to being of fundamental interest, improving our knowledge of the properties and behavior of adipocytes is essential for tackling the increasing prevalence of obesity in the developed world.

Zhou et al. now report that TAF7L—a gene that was previously thought to be involved only in the production of sperm cells—has two roles in the differentiation of stem cells to form adipocytes. Using a combination of cellular, biochemical, genetic and genomic techniques, they show that TAF7L interacts with PPARγ, an important adipocyte transcriptional regulator at enhancer sites on the genome to increase the transcription of genes that are involved in adipogenesis. They also show that TAF7L interacts with a general transcription factor called TBP (short for TATA-binding protein) at promoter sequences, again to increase the expression of genes involved in adipogenesis. Moreover, they show that the expression of TAF7L in myoblasts—precursor cells that usually become muscle cells—can induce the formation of fat cells rather than muscle cells. Furthermore, mice lacking TAF7L are lean compared to their normal littermates. A clearer understanding of the underlying causes of fat cell formation could lead to the development of new approaches for the treatment of obesity and associated diseases.

**DOI:** http://dx.doi.org/10.7554/eLife.00170.002

## Introduction

Adipose tissue plays a central role in energy homeostasis by acting as a major lipid storage site as well as an important endocrine tissue. Excess food intake vs energy expenditure invariably leads to obesity, an increasingly prevalent condition in modern societies. Obesity is, in turn, tightly associated with an elevated risk of type-2 diabetes, hypertension, cardiovascular disease, and certain cancers, posing serious public health issues and rapidly escalating costs of health care (Rosen and MacDougald, 2006; Rosen and Spiegelman, 2006).

Accumulation of adipose tissue mass results from increase in both adipocyte size and number. The size of adipocytes depends on the amount of stored lipids, while an increase in adipocyte number (adipogenesis) generally results from the expansion of adult precursor cells and subsequent differentiation, an underlying cause of obesity (Rosen and MacDougald, 2006). Consequently, there is great interest in dissecting the molecular mechanisms regulating adipogenesis and adipose biology.

During the past 20 years, numerous studies have focused on the formation of adipocytes using well-established in vitro cell models (Hollenberg et al., 1997; Tang et al., 2003; Zhang et al., 2004a; Tang et al., 2005; Tontonoz and Spiegelman, 2008). The utilization of these model cells identified key adipogenic transcriptional activators such as C/EBPα and PPARγ (Hu et al., 1995; Brun et al., 1996; Tang et al., 2003; Zhang et al., 2004b). Other transcription factors and cofactors, such as KLF15, KLF9, MED1, MED14, MED15, MED23 and TAF8, have also been reported to be involved in either adipocyte commitment or differentiation in model cell lines (Guermah et al., 2003; Mori et al., 2005; Matsumoto et al., 2007; Wang et al., 2009; Grøntved et al., 2010; Pei et al., 2011). More recently, genome-wide studies using a variety of techniques (microarray, FAIRE-seq, Quanttrx and mRNA-seq) have been utilized to screen for additional putative pro-adipogenic factors based on changes in either mRNA levels or chromatin states during adipogenesis (Gupta et al., 2011; Waki et al., 2011). Specifically, adipocyte-specific expression signatures and genome-wide binding maps of pro-adipogenic activators such as PPARγ, C/EBPα, and RXRα have been primarily determined from 3T3-L1 derived adipocytes. Other factors, including ZFP423, NF1 family proteins, and IRFs have been also implicated in adipogenesis (Eguchi et al., 2008; Mikkelsen et al., 2010; Schmidt et al., 2011; Waki et al., 2011; Boergesen et al., 2012).

However, it has become evident that the full complement of key transcriptional regulators that orchestrate adipogenesis remains to be illucidated. The diversity of factors and the mechanisms driving adipocyte formation and cellular differentiation in general remain a challenge. For example, several tissue-specific components of the core transcriptional machinery were recently found to play essential roles in directing cell type-specific programs of transcription and lineage-specific differentiation. The examples of these include TAF4b which was found to be a key component in the development of the mouse ovary and spermatogenesis (Freiman et al., 2001; Voronina et al., 2007), and TAF3 which was found to be required for mouse germ-layer differentiation (Liu et al., 2011). Additionally, a TRF3/TAF3 complex was found to be important for mouse myogenesis and zebrafsh hematopoiesis (Deato et al., 2008; Hart et al., 2009). As increasing numbers of cell type- and tissue-specific components of the core machinery become better characterized, a somewhat different notion of how traditional sequence–specific enhancer binding factors cooperate with selective tissue-specific core factors to drive lineage-specific transcription programs has emerged (D'Alessio et al., 2009). With respect to adipogenesis, the only hint of such a mechanism came from previous studies of TAF8, which was reported as a component of TFIID implicated in adipocyte formation using 3T3-L1 cells (Guermah et al., 2003). Whether TAF8 operates exclusively as a subunit of TFIID or participates in other molecular transactions, in addition to its in vivo function, remain unknown.

Here we found TAF7L, a paralogue of TATA binding protein associated factor 7, is highly enriched in differentiated C3H10T1/2 adipocytes and bona fide mouse WAT. We have utilized shRNA knockdown and gene knockout strategies to determine the role of TAF7L in adipocyte formation both in vitro and in vivo. In addition, we have explored the consequences of ectopically expressing TAF7L in C2C12 myoblasts to probe its reprogramming capabilities. Further, we carried out genome-wide mRNA-seq and ChIP-seq analysis to survey its functions in adipocyte differentiation. By using a combination of cellular, biochemical, genetic, and genomic approaches, our findings suggest that TAF7L plays an integral role in adipocyte gene expression by targeting enhancers as a cofactor for PPARγ and promoters as a component of the core transcriptional machinery, therefore providing new molecular insights into fat development that may prove useful for developing therapeutic strategies to treat obesity and its associated diseases.

## Results

### Elevated levels of TAF7L in differentiated adipocytes and WAT

**Figure 1.**
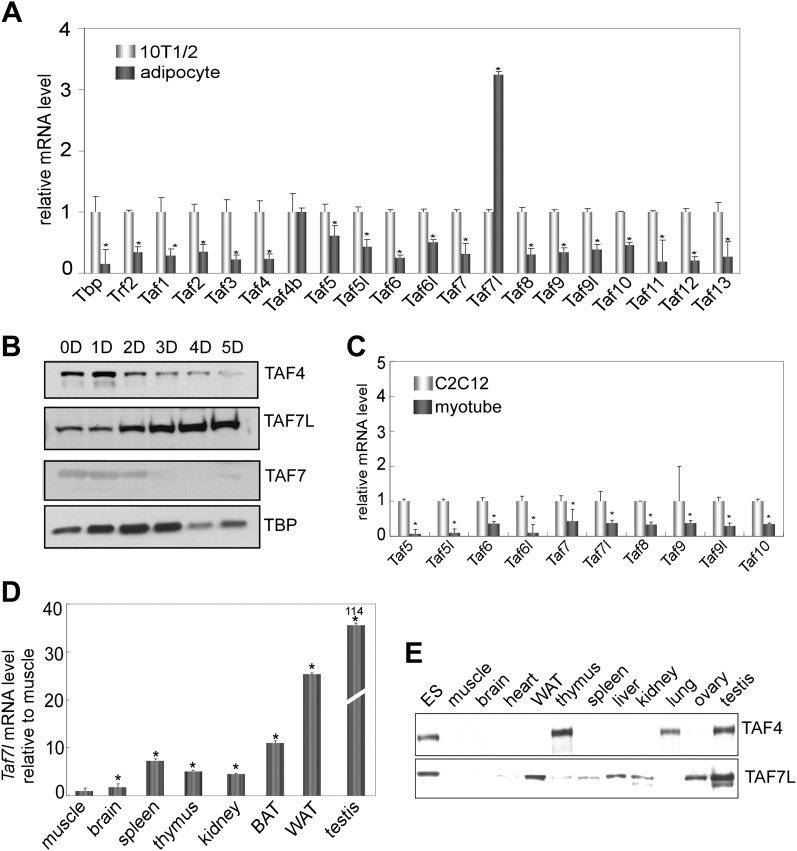
TAF7L is enriched in terminally differentiated adipocytes and bona fide WAT. (**A**) and (**B**) Expression of TAF7L and TFIID subunits prior to and 5 days (5D) post adipogenic induction of C3H10T1/2 cells as shown by RT-qPCR analysis (**A**) and by Western blot (**B**). (**C**) mRNA levels of TFIID subunits in C2C12 cells and myotubes. (**D**) *Taf7l* mRNA levels in different mouse tissues detected by RT-qPCR relative to muscle, whose expression level was assigned to 1 as the tissue displaying the lowest *Taf7l* mRNA levels. (**E**) Western blot analysis of mouse tissues with TAF4 and TAF7L antibodies. mRNA levels in (**A**) and (**C**) was assigned to 1 in C3H10T1/2 and C2C12 cells, mRNA levels in adipocytes and myotubes were compared with C3H10T1/2 and C2C12 cells respectively. *p<0.05, data is mean and s.e.m is from triplicates. RT-qPCR was normalized to the amount of total mRNA and Western blotting analysis was normalized to the amount of total protein. D, days; 10T1/2, C3H10T1/2 cells; ES, embryonic stem cell; BAT, brown adipose tissue; WAT, white adipose tissue. **DOI:** http://dx.doi.org/10.7554/eLife.00170.003

To explore the regulatory mechanisms directing adipocyte formation and function, we asked whether there were significant changes to the core promoter recognition components during adipogenesis similar to what had been observed during myogenesis (Deato and Tjian, 2007). In particular, we set out to determine whether and which TAF subunits of the prototypic core promoter recognition complex TFIID increase or decrease in expression during adipocyte differentiation. Consistent with the previous observations from 3T3-L1 adipogenesis studies (Guermah et al., 2003), our analysis of both protein and mRNA levels revealed that TBP and most of the canonical subunits of TFIID are down-regulated during C3H10T1/2 differentiation (Figure 1A,B). Surprisingly however, one subunit (TAF7L) previously reported to be a component of TFIID primarily in testis (Pointud et al., 2003; Cheng et al., 2007) was found to be significantly up-regulated in differentiated C3H10T1/2 adipocytes (Figure 1A,B and Figure 1—figure supplement 2A) and 3T3-L1 adipocytes (Figure 1—figure supplement 1A,C). Importantly, this enrichment appears specific for the adipogenesis process since the mRNA abundance of *Taf7l* is downregulated to levels comparable to those of other TAF subunits during myogenesis (Figure 1C). To exclude the possibility that *Taf7l* enrichment reflects a cell culture artifact of C3H10T1/2 adipogenesis, we compared *Taf7l* mRNA and protein levels in bona fide mouse tissue. In concordance with previous studies, *Taf7l* is most highly expressed in testis (Pointud et al., 2003) (Figure 1D,E). Importantly, *Taf7l* also shows significant expression in WAT and detectable expression in liver, spleen, brown adipose tissue (BAT) and kidney, but not in muscle or brain tissue (Figure 1D,E). By contrast, the expression of canonical TFIID subunits such as TAF4 is low in both WAT and muscle as expected (Figure 1E). Taken together, these data indicate that TAF7L is indeed enriched in differentiated C3H10T1/2 and 3T3-L1 adipocytes and bona fide WAT.

**Figure 1—figure supplement 1.**
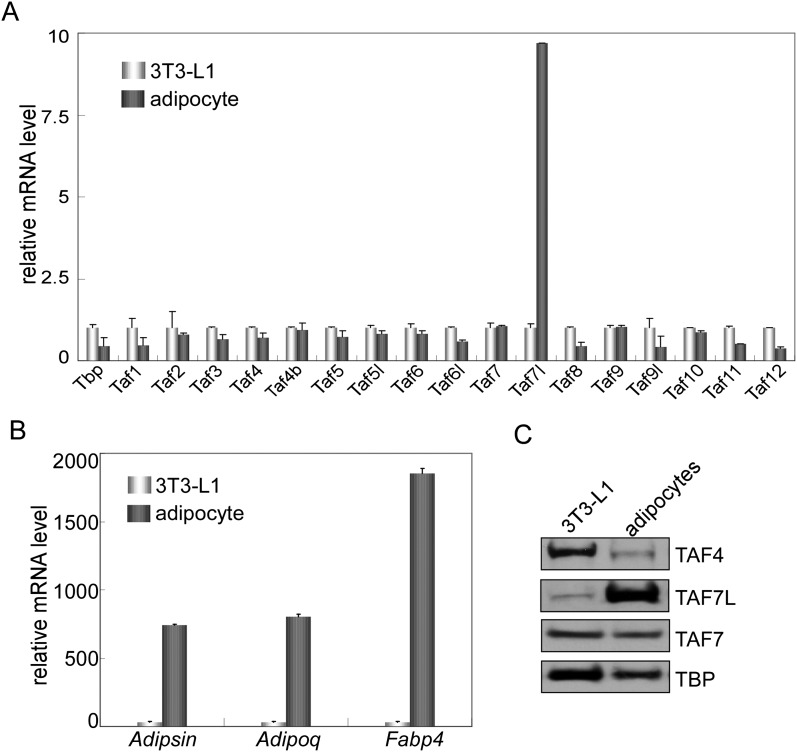
TAF7L is enriched in 3T3-L1 differentiated adipocytes. (**A**) Expression of *Taf7l* and TFIID subunits prior to and 7 days (7D) post adipogenic induction of 3T3-L1 cells as shown by RT-qPCR analysis (**A**) and by Western blot (**C**). (**B**) Gene expression of adipocyte marker genes *Adipsin*, *Adipoq* and *Fabp4* of 3T3-L1 adipocytes prior to and 7 days post adipogenic induction. mRNA levels in 3T3-L1 cells were assigned to 1, mRNA levels of each gene in 3T3-L1 adipocytes were compared to 3T3-L1 cells, data is mean from triplicates. **DOI:** http://dx.doi.org/10.7554/eLife.00170.004

**Figure 1—figure supplement 2.**
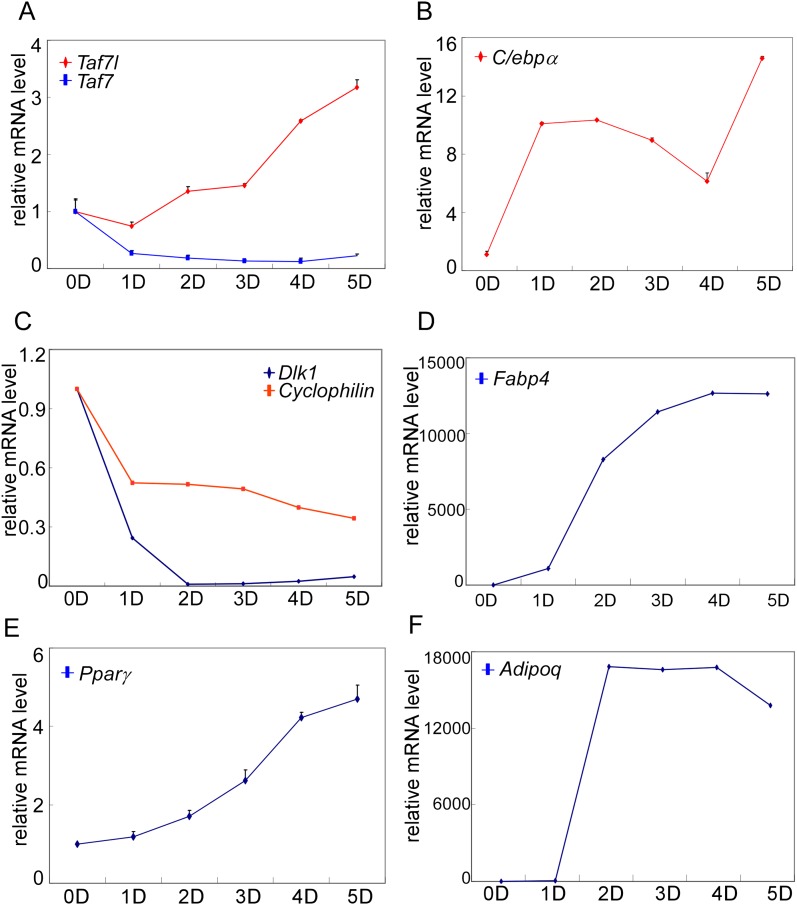
Gene expression analysis of C3H10T1/2 cells during adipogenesis. (**A**)–(**F**) Time course analysis by RT-qPCR analysis of *Taf7l* and *Taf7* (**A**), *C*/*ebpα* (**B**), *Dlk1* and *Cyclophilin* (**C**), *Fabp4* (**D**), *Pparγ* (**E**) and *Adipoq* (**F**) in C3H10T1/2 cells at 0D, 1D, 2D, 3D, 4D and 5D post adipogenic induction. D, days, mRNA levels in C3H10T1/2 cells at 0D were assigned to 1, mRNA levels of each gene at 0D, 1D, 2D, 3D, 4D, and 5D in C3H10T1/2 cells during adipogenesis were compared to 0D respectively, and data is mean from triplicates. **DOI:** http://dx.doi.org/10.7554/eLife.00170.005

These findings were surprising for several reasons. First, *Taf7l* had only been well documented to be critical for directing spermatogenesis in mice, and *Taf7l-*deficient mice show an impaired male fertility phenotype but no other defects were previously reported (Akinloye et al., 2007; Cheng et al., 2007). Second, although earlier studies of terminal differentiation implicated specific ‘atypical TAFs’ in, for example, skeletal muscle, ovary and testis formation (Freiman et al., 2001; Deato et al., 2008; D'Alessio et al., 2009), we did not anticipate *Taf7l* as a potential key player in adipogenesis. Instead, based on previous work, we expected that *Taf8* would emerge as the ‘cell-type specific’ TAF involved in adipogenesis (Guermah et al., 2003). However, we have found *Taf7l* to be up-regulated while *Taf8* mRNA is down-regulated upon induction of C3H10T1/2 or 3T3-L1 cells to form adipocytes (Figure 1A and Figure 1—figure supplement 1A). To explore this new finding, we set out to investigate the hitherto unrecognized potential role of *Taf7l* in adipogenesis.

### TAF7L is required for adipocyte-specific gene expression and differentiation

**Figure 2.**
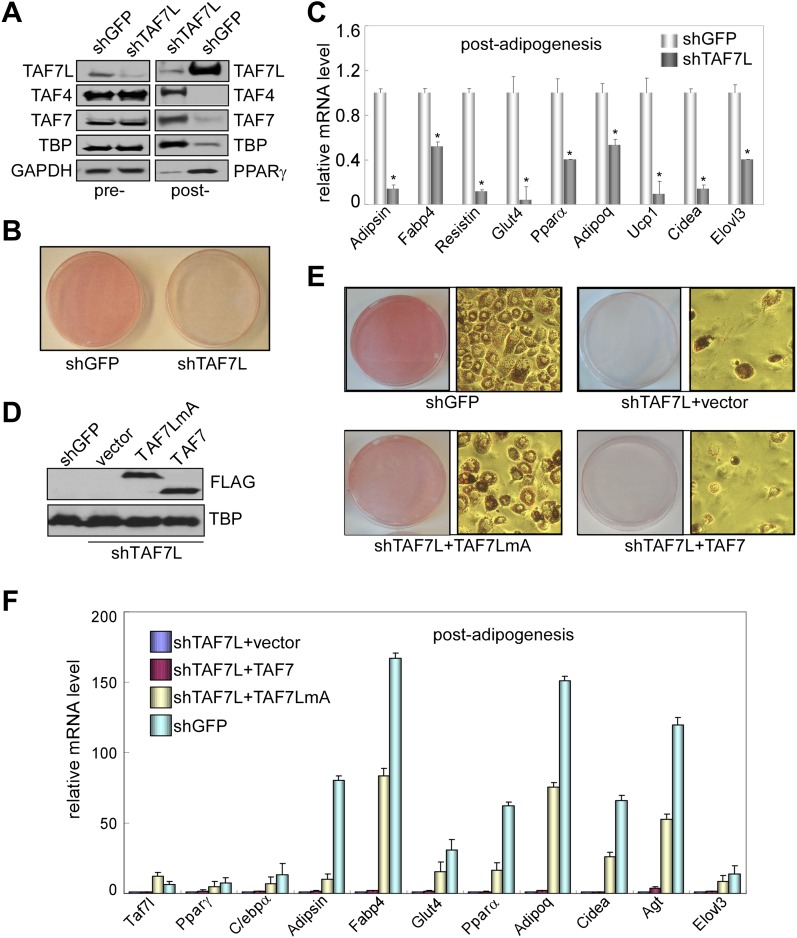
TAF7L is required for adipogenesis in vitro. (**A**) Western blot of TAF7L, TAF4, TAF7, TBP, GAPDH, and PPARγ protein levels in C3H10T1/2 cells expressing shRNA sequence against GFP as control (shGFP) or specifically against TAF7L (shTAF7L) pre- (left panel) and post-differentiation (right panel). GAPDH protein levels serve as a loading control. (**B**) Oil red O staining in 5 days differentiated C3H10T1/2 cells stably expressing either shGFP or shTAF7L. (**C**) mRNA levels of adipocyte-specific genes by RT-qPCR on differentiated shGFP or shTAF7L C3H10T1/2 cells from (**B**), mRNA levels in shGFP cells were assigned to 1, mRNA levels of each gene in shTAF7L cells were compared to shGFP cells, *p<0.05, data is mean and s.e.m is from triplicates. RT-qPCR was normalized to the amount of total mRNA. (**D**) Western blot with FLAG and TBP antibodies showing the expression of FLAG-TAF7LmA and FLAG-TAF7 in shTAF7L stably transfected C3H10T1/2 cells, TBP protein levels serve as a loading control. (**E**) Oil red O staining on shGFP or shTAF7L cells ectopically expressing FLAG, FLAG-TAF7 or FLAG-TAF7LmA 5 days post adipogenesis. (**F**) mRNA levels of adipocyte-specific genes by RT-qPCR in differentiated cells from (**E**), mRNA levels in shTAF7L + vector cells were assigned to 1, mRNA levels of each gene in shTAF7L + TAF7, shTAF7L + TAF7LmA and shGFP cells were compared to shTAF7L + vector cells, data is mean from triplicates. **DOI:** http://dx.doi.org/10.7554/eLife.00170.006

To assess whether *Taf7l* is required for adipogenesis, we first knocked down TAF7L expression in C3H10T1/2 cells and then induced adipogenesis. shTAF7L and control shGFP sequences were transfected into C3H10T1/2 cells to generate puromycin resistant stable TAF7L knockdown or shGFP control cell lines (Figure 2). As shown by Western blot, shTAF7L significantly reduced TAF7L protein levels both pre- and more dramatically post-adipogenesis while levels of canonical TFIID subunits remained largely unaltered in control and TAF7L knockdown pre-adipogenesis cultures (Figure 2A, pre-). Consistent with our previous observation in terminally differentiated cells, the protein levels of the canonical TFIID subunits become largely decreased in control post-adipogenesis cultures while cells that have been depleted of TAF7L and therefore blocked from differentiation show high levels of TFIID subunits. As expected, PPARγ levels increased in control shGFP cells post induction but showed markedly reduced levels in shTAF7L-treated cells suggesting that TAF7L may directly or indirectly regulate this key adipogenic transcription factor. Our results also suggest that shTAF7L efficiently reduced endogenous TAF7L levels and blocked adipogenesis without significantly affecting TFIID complex integrity (Figure 2A, post-). Next, C3H10T1/2 cells stably treated with shTAF7L and control shGFP were subjected to Oil red O staining to determine the efficiency of adipogenesis. Very few lipid-laden adipocytes formed in TAF7L depleted C3H10T1/2 cells, whereas over 98% of shGFP-treated cells differentiated into mature adipocytes. The few adipocytes that formed in shTAF7L-treated cells appeared smaller and exhibited abnormal morphology compared to untreated or shGFP-treated C3H10T1/2 adipocytes (Figure 2B,E). These results suggest that TAF7L knockdown largely compromised the adipogenic potential of C3H10T1/2 cells, thus functionally implicating TAF7L in adipocyte differentiation.

**Figure 2—figure supplement 1.**
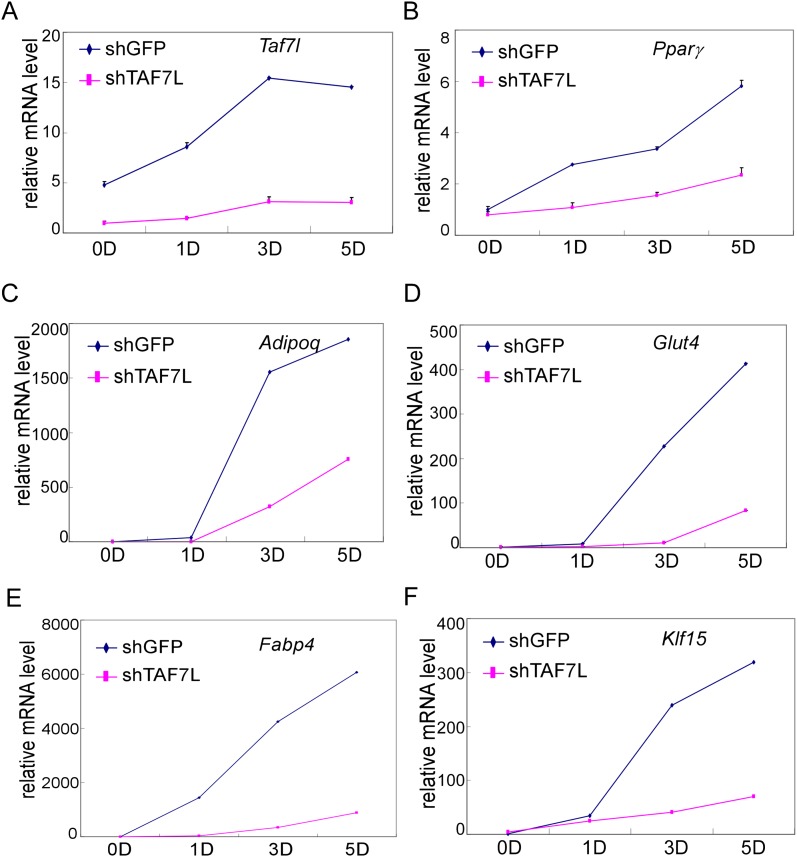
Gene expression analysis after TAF7L knockdown in C3H10T1/2 cells. (**A**)–(**F**) Time course of gene expression by RT-qPCR analysis of *Taf7l* (**A**), *Pparγ* (**B**), *Adipoq* (**C**), *Glut4* (**D**), *Fabp4* (**E**), and *Klf15* (**F**) in C3H10T1/2 cells stably treated with shGFP or shTAF7L sequences at 0D, 1D, 3D, and 5D post adipogenic induction. D, days; shGFP, control cells; shTAF7L, TAF7L knockdown cells. mRNA levels in shTAF7L-treated C3H10T1/2 cells at 0D were assigned to 1, mRNA levels of each gene at 0D, 1D, 3D, and 5D in both shGFP and shTAF7L-treated C3H10T1/2 cells during adipogenesis were compared to mRNA levels in shTAF7L-treated C3H10T1/2 cells at 0D respectively, data is mean from triplicates. **DOI:** http://dx.doi.org/10.7554/eLife.00170.007

To address the possibility that the observed adipogenic defects result from off-target effects of shTAF7L treatment, we carried out ‘rescue’ experiments by introducing either an empty vector, a shRNA-resistant vector TAF7LmA (Ding et al., 2008), or its control paralogue TAF7 into shTAF7L cells. The TAF7LmA expression vector contains two silent mutations in *Taf7l* cDNA which renders resistance to RNA-mediated silencing by shTAF7L. As revealed by Western blot, TAF7LmA is efficiently expressed in shTAF7L cells, similar to the TAF7 construct (Figure 2D). Next, we induced adipogenesis followed by Oil red O staining 5 days post induction. Our results indicate that introduction of TAF7LmA restored adipocyte formation (Figure 2E) and elevated expression of adipocyte-specific genes compared to vector control in shTAF7L cells (Figure 2F). In contrast, overexpression of TAF7 failed to restore the adipogenic defects caused by the loss of TAF7L (Figure 2E). Collectively, these results support the notion that TAF7L is likely an important player in adipogenesis, at least in the C3H10T1/2 cell differentiation model.

**Figure 3.**
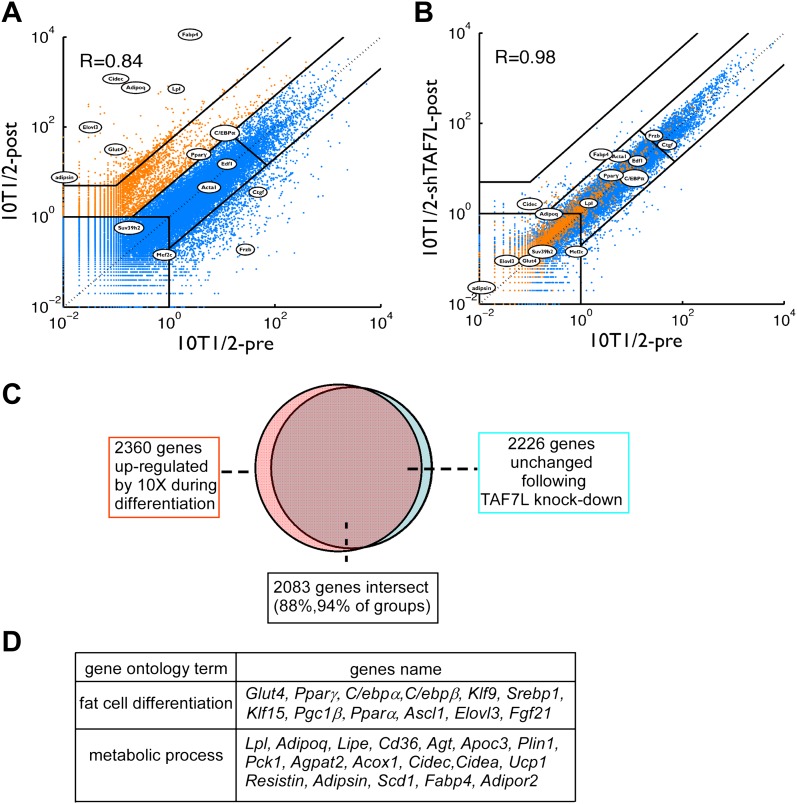
TAF7L is required for the expression of adipocyte-specific genes. (**A**) and (**B**), mRNA-seq data on gene expression of C3H10T1/2 cells pre- (horizontal axis) and post-adipogenesis (vertical axis) (**A**); mRNA-seq data on gene expression in C3H10T1/2 cells pre-adipogenesis (horizontal axis) and C3H10T1/2 treated with shTAF7L post-adipogenesis (vertical axis) (**B**). Orange dots in (**A**) mark genes upregulated during adipogenesis; blue dots in (**A**) mark genes unchanged or downregulated during adipogenesis. Circled genes were tested individually in RT-qPCR analysis. R indicates the correlation of the expression programs between two compared cells (10T1/2-post vs 10T1/2-pre in (**A**), 10T1/2-shTAF7L-post vs 10T1/2-pre in (**B**)). (**C**) TAF7L knockdown blocks the upregulation of the adipocyte-specific genes which occurs during normal adipogenesis, pink circle represents 2360 genes upregulated in 10T1/2-post by 10-fold (10×) from (**A**); orange circle represents 2226 genes unchanged in 10T1/2-shTAF7L-post (**B**) compared to (**A**), 2083 genes in the overlapping intersect region account for 88% of total upregulated 10× genes in (**A**). (**D**) List of gene ontology analysis hits showing a few typical adipocyte genes involved in fat cell differentiation and metabolic processes. **DOI:** http://dx.doi.org/10.7554/eLife.00170.008

To identify the full range of genes regulated by TAF7L in adipocyte differentiation, we performed mRNA-seq to profile global gene expression patterns in C3H10T1/2 cells prior to (10T1/2-pre) and after adipogenesis (10T1/2-post) (Figure 3A). Next, we verified our mRNA-seq results by single gene RT-qPCR assays for a handful of well-characterized adipocyte-specific genes such as *Fabp4*, *Glut4*, *Adipsin*, *Lpl*, as well as control genes such as *Mef2c* and *Frzb* (data not shown); these results confirmed high concordance between the RT-qPCR assays and the genome-wide mRNA-seq data, although RT-qPCR generally gave 3- to 4-fold higher sensitivity compared to mRNA-seq. We also surveyed a set of 2360 genes upregulated by 10-fold or more in C3H10T1/2 cells post- vs pre-differentiation (Figure 3); this analysis identified nearly all the well characterized adipocyte-specific genes including a large proportion of genes involved in adipocyte development and function (Figure 3D).

Next, we measured gene expression programs in differentiated C3H10T1/2 cells after depletion of TAF7L (10T1/2-shTAF7L-post). Importantly, genes normally up-regulated following adipogenesis (Figure 3A, shown in orange) showed similarly low expression levels in induced shTAF7L cells as in pre-adipocytes (Figure 3B, orange). Strikingly, 2083 out of 2360 genes (88%) that are highly upregulated during adipocyte differentiation failed to be induced upon adipogenic induction in the absence of TAF7L (Figure 3B,C). RT-qPCR analysis of several representative marker genes confirmed that TAF7L knockdown dramatically reduced their mRNA levels compared to control shGFP in post-adipogenesis cells (Figure 2C and Figure 2—figure supplement 1). These data suggest that the induction of adipocyte-specific genes is markedly compromised in the absence of TAF7L. Moreover, the overall transcriptional profile of differentiated shTAF7L-treated C3H10T1/2 cells (shTAF7L-post) largely matched the expression levels of pre-differentiated C3H10T1/2 cells (10T1/2-pre) (R = 0.98) (Figure 3B); while mature adipocytes (10T1/2-post) is distinct (R = 0.84) (Figure 3A). Thus, loss of TAF7L in C3H10T1/2 cells severely impaired its adipogenic potential rendering shTAF7L-treated cells in an undifferentiated state (Figures 2B and 3A,B). These findings were also confirmed using a different shTAF7L construct and RT-qPCR analysis (data not shown). Taken together, these results strongly implicate TAF7L in potentiating efficient adipogenesis of C3H10T1/2 cells by serving as an important regulator of adipocyte-specific gene expression.

### *Taf7l* is required for WAT development in vivo

**Figure 4.**
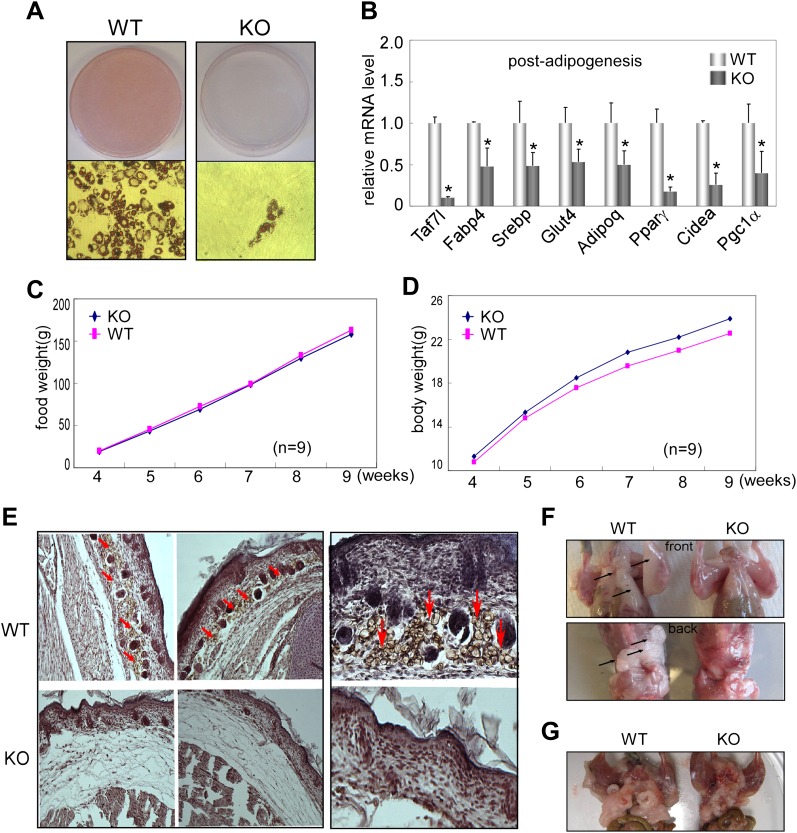
*Taf7l* is required for WAT development in vivo. (**A**) Oil red O staining to detect mature adipocytes from 5 day differentiated primary fibroblasts derived from adipose tissue of wild-type (WT) and *Taf7l*-deficient mice (KO). (**B**) mRNA levels of adipocyte-specific genes by RT-qPCR on WT and *Taf7l* KO primary fibroblasts post differentiation from (**A**), mRNA levels in WT cells were assigned to 1, mRNA levels of each gene in *Taf7l* KO cells were compared to WT cells, *p<0.05, data is mean and s.e.m is from triplicates. RT-qPCR was normalized to the amount of total mRNA. (**C**) Average food intake of WT and KO mice from week 4 to week 9 after birth. n = 9. (**D**) Average body weights of WT and KO littermates from week 4 to week 9 after birth, n = 9. (**E**) H&E and FABP4 antibody stain subcutaneous fat cells in E18.5 WT and *Taf7l* KO embryos, left panel magnification, ×5; right panel magnification, ×20; red arrows indicate fat cells stained by FABP4. (**F**) *Taf7l* KO mice exhibits less fat tissue than WT littermate. Shown are representative photographs of 1-month-old mice with skin removed from both front and back views. (**G**) *Taf7l* KO mouse exhibits less fat formation than WT littermate. Shown is a representative photograph of 4-month-old mouse with skin removal. **DOI:** http://dx.doi.org/10.7554/eLife.00170.009

To assess *Taf7l* function in adipogenesis in vivo, we first isolated primary adipocyte fibroblasts from WAT of *Taf7l* knockout (KO) mice and littermate controls (WT) and tested their ability to undergo adipogenesis. As revealed by Oil red O staining, *Taf7l* KO fibroblasts produced very few, if any, lipid-filled adipocytes compared to WT cells in response to adipogenic induction (Figure 4A). RT-qPCR analysis confirmed that ablation of *Taf7l* also suppresses the upregulation of adipocyte-specific genes during differentiation (Figure 4B). These results indicate a requirement for *Taf7l* in adipocyte differentiation of primary adipocyte fibroblasts, consistent with our results from C3H10T1/2 cells. As expected, loss of *Taf7l* caused no obvious change in mRNA or protein levels of other TFIID subunits (data not shown), suggesting that deletion of *Taf7l* is unlikely to affect TFIID integrity or function in vivo, in agreement with previous observations that *Taf7l*-deficient mice appear normal except for germ cell developmental defects (Cheng et al., 2007).

To determine the influence of *Taf7l* KO in early fat development in vivo, we next examined the adipose formation in E18.5 embryos of WT control and *Taf7l* KO littermates. We utilized haematoxylin and eosin (H&E) staining on transversal sections of the interscapular region to visualize the overall structure of skin and underlying tissue including subcutaneous fat, connective tissue and muscle. In particular, we used FABP4 antibody staining to localize developing subcutaneous adipose tissue. We observed that FABP4^+^ lipid-laden cells are significantly diminished in the subcutaneous layer of *Taf7l* KO mice compared to WT littermate controls with a concomittant increase in layers of connective-like tissue under the skin of *Taf7l* KO mice (Figure 4E). These results suggest that loss of *Taf7l* impairs WAT development in the later stages of embryogenesis (Gupta et al., 2010). It also appears that in *Taf7l*-deficient mice, there may be an imbalance in mesodermal-derived lineages as revealed by the appearance of a thicker layer of subcutaneous connective-like tissue.

We next examined the formation of WAT in 1-month and 4-month-old *Taf7l* KO and control WT littermates. The results revealed that 5 out of 12 *Taf7l* KO mice exhibited a noticeable reduction in both their subcutaneous and abdominal white fat pads compared to control WT animals (Figure 4F,G), while both groups consumed similar amounts of food and have similar growth curves (Figure 4C,D). Taken together, our observations with isolated primary adipose fibroblasts, E18.5 embryos, and fat pads from young mice are consistent with the notion that *Taf7l* likely functions in potentiating mouse WAT development.

### Ectopic expression of TAF7L transdifferentiates C2C12 myoblast

**Figure 5.**
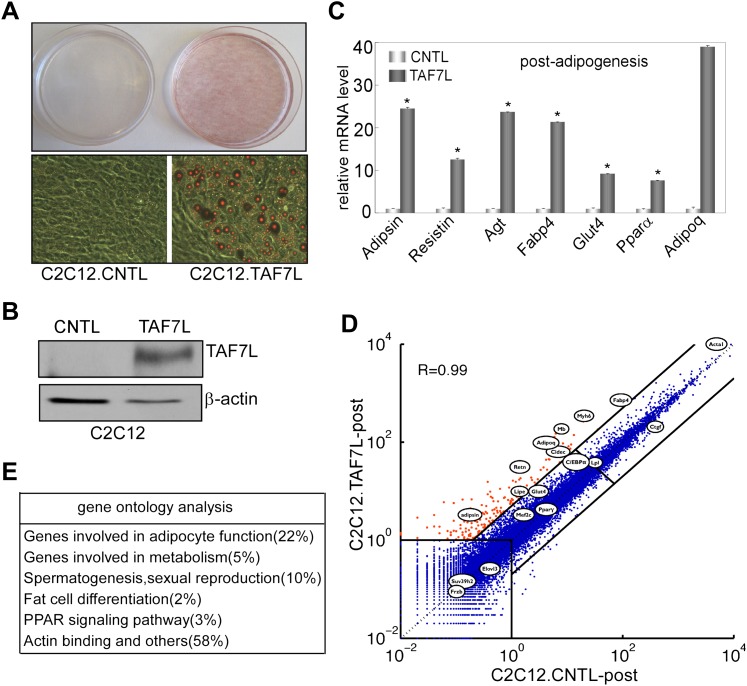
Ectopic expression of TAF7L transdifferentiates C2C12 myoblasts into adipocytes under adipogenic induction. (**A**) C2C12 myoblasts expressing empty vector (C2C12.CNTL) or TAF7L (C2C12.TAF7L) were stained with Oil red O 5 days after inducing adipogenesis. (**B**) Western blot analysis on ectopic expression levels of FLAG-TAF7L in C2C12.7L and C2C12.CNTL cells, β-actin protein level is served as a loading control. CNTL, C2C12.CNTL; TAF7L, C2C12.TAF7L. (**C**) mRNA levels of adipocyte marker genes are measured by RT-qPCR in C2C12.TAF7L cells compared with C2C12.CNTL cells 5 days post adipogenesis, mRNA levels of genes in C2C12.CNTL cells were assigned to 1. *p<0.05, data is mean and s.e.m is from triplicates. RT-qPCR was normalized to the amount of total mRNA. (**D**) mRNA-seq analyzes genes activated by TAF7L in C2C12.TAF7L compared to C2C12.CNTL post adipogenesis. Red dots represent genes upregulated in C2C12.TAF7L-post cells; blue dots represent genes unaltered or downregulated in C2C12.TAF7L-post cells compared to C2C12.CNTL-post cells after adipogenic induction. (**E**) Major gene functional groups from genes activated above fivefold by TAF7L in C2C12 cells post adipogenic induction through gene ontology analysis. **DOI:** http://dx.doi.org/10.7554/eLife.00170.010

A complementary strategy to probe the capacity of transcription factors to influence specific differentiation pathways involves the ‘reprogramming of cell fate’. For instance, transdifferentiation of C2C12 myoblasts into adipocytes by ectopic expression of PPARγ and/or C/EBPa under adipogenic permissive conditions helped establish these sequence-specific enhancer binding factors as key regulators of adipogenesis (Tontonoz et al., 1994a, 1994b; Hu et al., 1995; Kajimura et al., 2009). Therefore, we tested the adipogenic function of TAF7L by an analogous ‘gain of function’ approach with forced introduction of TAF7L or control vector into C2C12 myoblasts. First, we generated TAF7L-expressing (C2C12.TAF7L) or control C2C12 (C2C12.CNTL) stable cell lines by transfecting either FLAG-TAF7L or empty vector followed by neomycin selection. As detected by FLAG antibody through Western blot analysis, C2C12.TAF7L stable cells achieved modestly elevated expression levels of FLAG-TAF7L protein (Figure 5B). Similarly, C2C12.TAF7L cells express roughly eightfold higher *Taf7l* mRNA levels than C2C12.CNTL cells (Figure 5—figure supplement 1A). Next, we treated both C2C12.TAF7L and C2C12.CNTL stable cell lines with the four standard adipogenic inducers for 5 days and then applied Oil red O staining. A large proportion of C2C12.TAF7L cells developed into lipid-laden cells while no detectable C2C12.CNTL cells produced lipid droplets (Figure 5A). Gene expression analysis by RT-qPCR confirmed that C2C12.TAF7L cells have markedly increased mRNA levels of a subset of adipocyte-specific genes including *Adipsin*, *Resistin*, *Pparγ*, *C*/*EBPα*, *Adipoq* and *Fabp4* relative to C2C12.CNTL cells post differentiation (Figure 5C and Figure 5—figure supplement 1B,C,E,F). By contrast, myoblast-gene *Myf5* is downregulated in C2C12.TAF7L cells prior to and post adipogenic induction (Figure 5—figure supplement 1D). Furthermore, we performed mRNA-seq on differentiated C2C12.TAF7L and C2C12.CNTL cells and these genome-wide expression studies revealed that indeed, a number of adipocyte-specific genes become highly upregulated in C2C12.TAF7L cells compared to C2C12.CNTL cells post adipogenesis (Figure 5D). Gene ontology analysis of genes upregulated fivefold or more in differentiated C2C12.TAF7L cells vs C2C12.CNTL cells indicated that ∼32% of these genes are involved in either adipocyte differentiation or function. Notably, in accordance with the role of *Taf7l* in spermatogenesis that was reported previously, ∼10% of these up-regulated genes are involved in spermatogenesis and sexual reproduction (Figure 5E). Taken together, these results indicate that ectopic expression of TAF7L in C2C12 myoblasts, even at modestly elevated levels, is capable of inducing upregulation of *Taf7l* itself and other important adipogenic transcription factors including *Pparγ* and *C*/*ebpα* (Figure 5—figure supplement 1A–C) thereby reprogramming a significant portion of C2C12 cells into adipocytes upon induction (Figure 5—figure supplement 1E,F), providing further evidence for TAF7L as a pro-adipogenic regulator.

**Figure 5—figure supplement 1.**
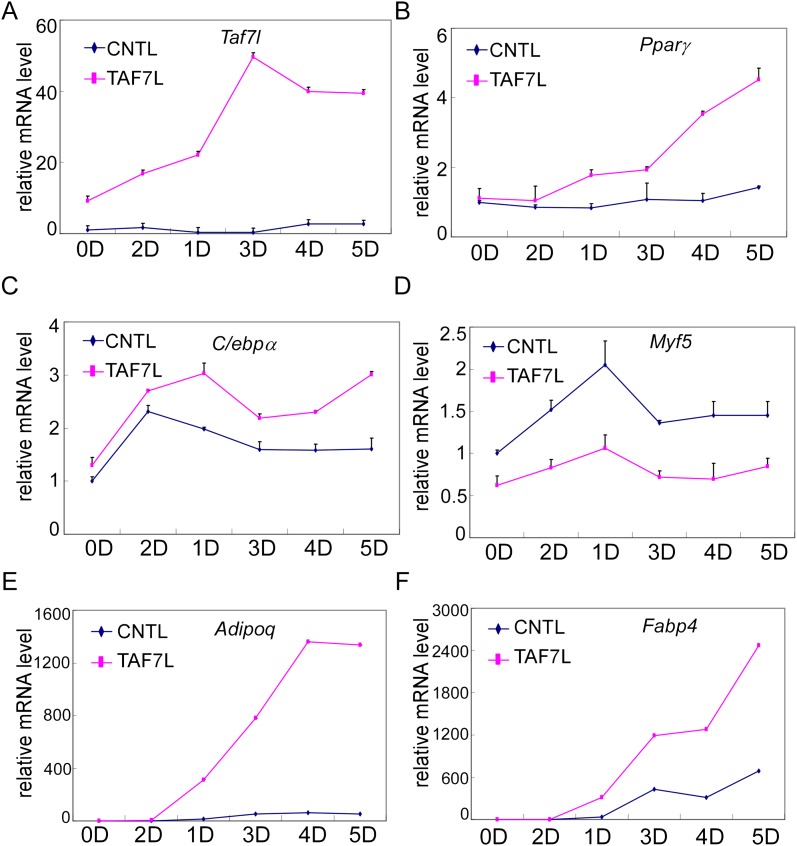
Gene expression analysis of TAF7L-expressing C2C12 cells. (**A**)–(**F**) Time course of gene expression by RT-qPCR analysis of *Taf7l* (**A**), *Pparγ* (**B**), *C/ebpα* (**C**), *Myf5* (**D**), *Adipoq* (**E**), and *Fabp4* (**F**) in C2C12.CNTL and C2C12.TAF7L cells at 0D, 1D, 2D, 3D, 4D and 5D post adipogenic induction. D, days; CNTL, C2C12.CNTL; TAF7L, C2C12.TAF7L. mRNA levels in C2C12.CNTL cells at 0D were assigned to 1, mRNA levels of each gene at 0D, 1D, 2D, 3D, 4D, and 5D in both C2C12.CNTL and C2C12.TAF7L cells during adipogenesis were compared to mRNA levels in C2C12.CNTL cells at 0D respectively, data is mean from triplicates. **DOI:** http://dx.doi.org/10.7554/eLife.00170.011

### TAF7L occupancy at a majority of adipocyte-specific genes

**Figure 6.**
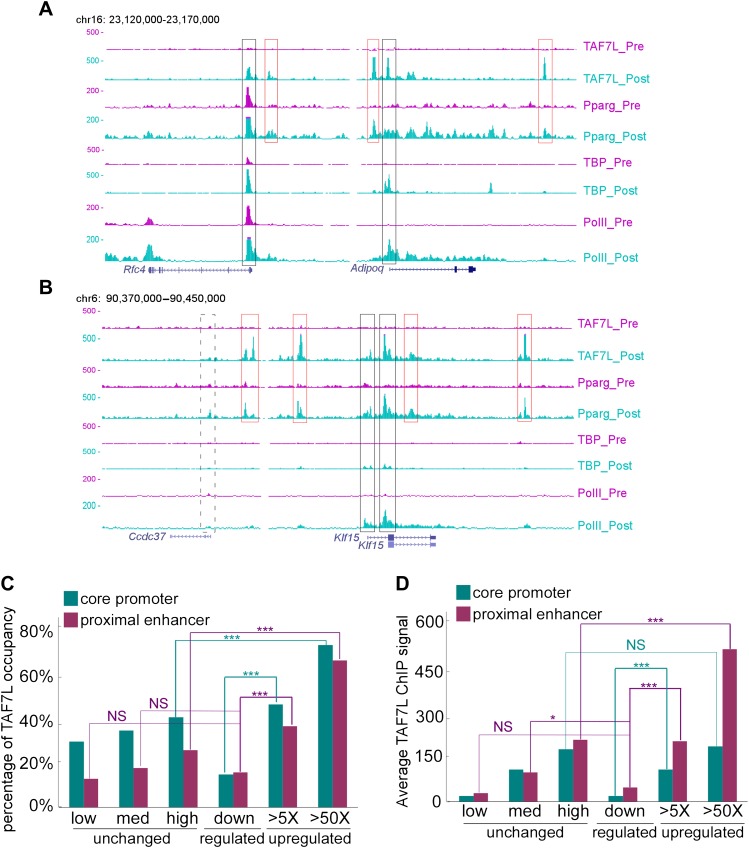
TAF7L binds strongly on the majority of genes upregulated during adipogenesis. (**A**) Read accumulation for eight ChIP-seq datasets including TAF7L, PPARγ, TBP and Pol II before (_pre) and after (_post) adipocyte differentiation at the *Rfc4* and *Adipoq* gene loci. (**B**) The same as in (**A**) at the *Ccdc37* and *Klf15* gene loci. Vertical axis is 0–500 reads for all factors, co-localized peaks were marked with boxes, black boxes indicate promoters and red boxes indicate enhancers, solid lines denote active genes and dashed lines denote inactive gene. (**C**) Frequency (vertical axis) of TAF7L occupancy on gene expression groups (horizontal axis) including unchanged (low, med, high) (three blue dots regions from left-bottom to right-top in Figure 3A), downregulated (blue dots in left-bottom region in Figure 3A), and upregulated (>5×, >50×, two orange dots regions from lower to higher in Figure 3A). (**D**) Average TAF7L binding signal strength (vertical axis) on the core promoters (500 bp from TSS) and proximal enhancers (500 bp to 5 kb from TSS) of three major gene expression groups as in (**C**). (Regular t-test for (**C**) and (**D**), NS is no significant, *p<0.05, ***p<0.001). **DOI:** http://dx.doi.org/10.7554/eLife.00170.012

To explore the potential mechanism by which TAF7L functions during adipogenesis, we took advantage of chromatin immunoprecipitation combined with deep sequencing (ChIP-seq) to map TAF7L, TBP, and Pol II binding profiles genome-wide prior to and after adiopgenesis. Mapped ChIP tags were analyzed by intersecting MACS and Grizzly Peak algorithms (Zhang et al., 2008; Feng et al., 2011; Harrison et al., 2011) to identify binding regions for each factor. In agreement with the low concentration of TAF7L shown in Western blots prior to differentiation (Figure 1A), only one significant peak was identified in C3H10T1/2 cells compared to 18,672 significant TAF7L binding peaks detected after adipocyte formation. At the same time, we found comparably large numbers of peaks for TBP (12,883 and 14,587) and Pol II (14,502 and 11,424) in pre- and post-differentiation C3H10T1/2 cells. As an example of our TAF7L ChIP-seq data, the profiles of two typical adipocyte-specific genes *Adipoq* and *Klf15*, a general highly expressed gene *Rfc4*, and a nonactive gene *Ccdc37* are shown in Figure 6A,B. The expression level of each gene before and after differentiation can be deduced from the enrichment levels of Pol II.

We split the genome into three groups representing unchanged, downregulated, and upregulated (>5×, >50×) genes based on their expression pattern and levels before and after differentiation based on mRNA-seq data. Analysis of our ChIP-seq and mRNA-seq data suggests that among all active genes in adipocytes, TAF7L binds to >65% of adipocyte-specific genes that are highly upregulated (>50×) during adipogenesis while binding much less frequently at both core promoters (<500 bp from TSS) and proximal enhancer regions (500 bp to 5 kb from TSS) near genes unaltered or downregulated during differentiation (Figure 6C). Next we compared the expression levels of genes in adipocytes to the binding intensity of TAF7L and found a strong positive correlation in the three expression groups. For instance, examination of 3,468 TAF7L peaks, representing 20% of the total peaks located at proximal enhancers revealed that the average TAF7L binding strength on genes upregulated >50× is twice as strong as on genes induced between 5–50× in adipocytes relative to C3H10T1/2 cells, and eight times stronger than on genes down-regulated following adipogenesis (Figure 6D), suggesting that TAF7L binding frequency and strength is highly correlated with upregulated genes during adipogenesis.

**Figure 7.**
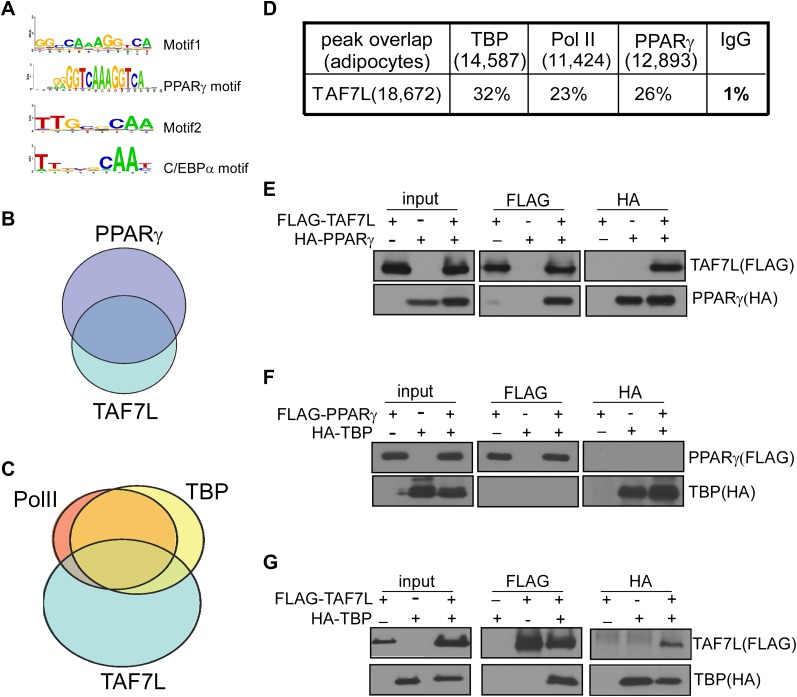
TAF7L colocalizes and associates with PPARγ and TBP. (**A**) Two top motifs (motif 1 and motif 2) were found in TAF7L binding sites. Motif1 p<2e-20) matches with PPARγ binding motif and motif 2 p<3e-10) matches with C/EBPα binding motif. (**B**) Overlap of PPARγ peaks with TAF7L peaks in adipocytes, each circle represents the total peaks from ChIP-seq for a factor and the overlapped region represents the common binding peaks of the factors. (**C**) Similar as in (**B**); Pol II, TBP and TAF7L peaks from ChIP-seq overlap with each other in adipocytes. (**D**) Table showed the total peak numbers of each factor in adipocytes from ChIP-seq and the percentage of genome-wide peak overlapping between TAF7L and PPARγ, Pol II, TBP, IgG control. (**E**) FLAG tagged TAF7L, HA tagged PPARγ were overexpressed in 293T cells, immunoprecipitations were performed on both FLAG and HA antibodies and followed by Western blotting with FLAG and HA antibodies. (**F**) The same procedures were performed on FLAG tagged PPARγ and HA tagged TBP. (**G**) The same procedures were performed on FLAG tagged TAF7L and HA tagged TBP as in (**E**). **DOI:** http://dx.doi.org/10.7554/eLife.00170.013

By mapping the genomic binding sites, we found that both TBP and Pol II display greater than 63% occupancy at transcriptional start sites (TSS) of highly expressed genes in both undifferentiated C3H10T1/2 cells and adipocytes, consistent with their roles in mediating global and general transcription functions. Interestingly, comparing the binding regions of TAF7L, TBP and Pol II in adipocytes revealed that TAF7L only partially (30%) colocalizes with TBP and Pol II at a subset of promoters, while a greater proportion (45%) of TAF7L peaks localizes to enhancer regions where TBP and Pol II are generally not found (Figure 7B,C). This surprising finding suggests that TAF7L may function via additional mechanisms other than as a subunit of canonical TFIID in regulating adipocyte differentiation.

### TAF7L co-localizes with PPARγ genome-wide

To determine mechanisms by which TAF7L operates via its occupancy at enhancers, we probed its potential association with adipocyte-specific enhancer binding transcription factors. First, we applied an unbiased genome-wide approach to identify sequence-specific transcription factors that could interact with TAF7L. We analyzed the sequences surrounding TAF7L binding sites and identified several DNA consensus sequence motifs enriched in TAF7L peaks. Next, we compared these motifs with all known sequence-specific recognition elements of transcription factors, which led to the identification of several binding motifs of well-known adipogenic transcription factors including PPARγ and C/EBPγ (Ge et al., 2008) (Figure 7A).

We then used ChIP-seq to directly map the genome-wide binding profiles of PPARγ and detected 4,121 and 12,893 significant peaks for PPARγ in C3H10T1/2 cells pre- and post-differentiation, respectively. We then validated our ChIP-seq data by comparing our new data with published ChIP-seq data for PPARγ, C/EBPα, and RXRα in 3T3-L1 derived adipocytes 6 days post-differentiation (Mikkelsen et al., 2010; Schmidt et al., 2011; Waki et al., 2011). This extensive analysis revealed that the majority of PPARγ binding sites we mapped overlap with those previously reported although some differences in specific binding sites were observed, likely due to inherent differences between 3T3-L1 and C3H10T1/2 cells. A direct comparison of TAF7L and PPARγ genome-wide binding profiles revealed that 26% of TAF7L peaks were co-occupied by PPARγ and reciprocally, 37% of PPARγ peaks were co-bound by TAF7L (Figure 7B,D). Similarly, TAF7L binds to 20% of RXRα bound loci genome-wide. Moreover, TAF7L also co-localizes with 25% of C/EBPα binding and vice versa. Collectively, these findings provide indirect evidence for a functional association between TAF7L and adipogenic activators PPARγ, RXRα and C/EBPα in adipocytes. Indeed, the relationship between TAF7L, PPARγ, C/EBPα and TBP/Pol II at a genome-wide scale suggests that TAF7L might also functions as an enhancer-associated co-activator that connects adipogenic activators with the core promoter recognition machinery to potentiate adipocyte differentiation.

### TAF7L interacts with PPARγ and TBP/TFIID

Prompted by the extensive co-localization between TAF7L, TBP and PPARγ, we set out to examine the potential physical association between these factors. First, we constructed TAF7L, TBP and PPARγ with either FLAG or HA Tags for expression in 293T cells in pair-wise combinations. Next, we performed co-immunoprecipitations (co-IP) between PPARγ, TAF7L and TBP with either FLAG or HA antibodies, followed by Western blot analysis to detect the FLAG- or HA-tagged proteins. Intriguingly, these co-IP assays showed that PPARγ can efficiently pull down TAF7L and vice versa with (data not shown) or without the addition of PPARγ ligand rosiglitozone (Figure 7E); by contrast, PPARγ was unable to co-IP TBP (Figure 7F). As expected, TAF7L and TBP can pull down each other reciprocally (Figure 7G), which is consistent with previous observations (Pointud et al., 2003). To confirm that the association between TAF7L and PPARγ or TBP is direct and not mediated via DNA/chromatin interactions, we included benzonase treatment in our co-IP assays. Eliminating DNA in these co-IP experiments did not alter the binding interactions we observed between TAF7L and PPARγ or TBP (data now shown). As expected, over-expression of TAF7L in C3H10T1/2 cells and adipocytes allows co-IP of other endogenous TFIID subunits including TBP and TAF4 (data not shown), suggesting that some tagged-TAF7L can integrate into native TFIID complexes. Taken together, these protein:protein binding assays suggest that TAF7L can physically associate with both PPARγ and TBP/TFIID either directly or indirectly via presently unidentified protein factors. These studies provide a potential mechanism by which TAF7L may serve as a cofactor linking specific adipogenic activators, proximal enhancers and the core transcription apparatus.

## Discussion

**Figure 8.**
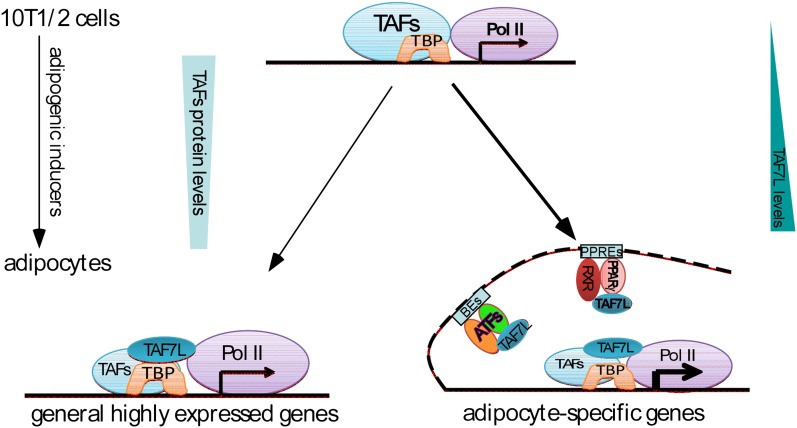
Dual functions of TAF7L in adipocyte differentiation. TAF7L expression is enriched during C3H10T1/2 MSCs adipocyte differentiation while other TFIID subunits (TAFs) decrease in expression. TAF7L regulates adipogenesis by associating with TBP as a component of adipocyte TFIID complex at promoters and with PPARγ or other adipocyte transcriptional factors (ATFs) as a cofactor at enhancers on adipocyte-specific genes, providing the mechanisms of its dual roles during differentiation. General highly-expressed genes are those with high expression before and after adipocyte differentiation include a portion of housekeeping genes; adipocyte-specific genes are those required for adipocyte differentiation and highly upregulated during adipocyte differentiation. TAFs,TBP-associated factors; ATFs, adipocyte transcriptional factors; BEs, binding elements. **DOI:** http://dx.doi.org/10.7554/eLife.00170.014

It is well-documented that the adult human body contains cells residing in the adipose tissue, referred to as Adipose-derived Stem Cells (ASCs) (DeLany et al., 2005; Feve, 2005; Gonzalez, 2005; Gerlach et al., 2012). ASCs resemble mesenchymal stem cells (MSCs) in terms of their ability to differentiate into multiple lineages including adipocytes, myotubes, osteocytes, and cartilage under appropriate developmental cues (Gornostaeva et al., 2006). Given that increased numbers of adipocytes, a major underlying cause of obesity, are primarily derived from MSCs and/or ASCs (Bowers and Lane, 2008), we chose C3H10T1/2 MSCs as our cell culture model system for studying adipogenesis in large measure because MSCs efficiently recapitulate aspects of adipocyte differentiation and in vivo fat development. Using this MSC culture model as well as *Taf7l* KO mouse model for our in vitro and in vivo studies, we unexpectedly identified *Taf7l* as a key regulator of adipogenesis; adding a new piece of the molecular puzzle to the critically important regulators of fat development in mammalian organisms. We found the effect of *Taf7l* in adipogenesis to be quite robust wherein its loss led to extensive down-regulation of genome-wide adipocyte-specific gene expression in cell culture and defects in WAT development in vivo. We are particularly intrigued by the manner in which TAF7L seems to operate–serving both as an integral component of TFIID at the core promoter and as a key co-activator interacting directly with PPARγ or other adipocyte-specific transcriptional factors (ATFs) at proximal enhancers of adipocyte-specific genes on genome-wide scale (Figure 8). Thus, a hitherto unrecognized cell-type selective core regulator with an apparent dual mechanism of action has been identified that influences the pro-adipogenic transcriptional control network. It is conceivable that TAF7L and associated regulatory factors in this newly discovered pathway may reveal potentially useful therapeutic drug targets to combat obesity and its related diseases.

It came as a surprise to find *Taf7l* playing such an essential role in adipogenesis because previous studies had primarily reported a specific role of *Taf7l* in spermatogenesis (Vernon et al., 1986; Armstrong et al., 1994; Hashmi et al., 2011). Indeed, even after being clued into the potential contribution of *Taf7l* in fat tissue development, our analysis of *Taf7l* KO mice mainly revealed defects in WAT formation at certain stages of development and there were no gross, easily-observable morphological abnormalities in young animals except when the underlying fat pads were dissected for direct inspection to reveal the partial penetrance of the lean phenotype. Also, previous studies had no reason to examine the expression of *Taf7l* in adipose tissue because *Taf7l* mRNA and protein levels are relatively low in adipose tissue compared with testis. We also note that although TAF7L protein levels become highly elevated when MSCs differentiate into adipocytes, its mRNA levels increase only modestly (∼2×) compared to typical adipocyte marker genes such as *Fabp4* and *Glut4* during adipogensis (Kajimura et al., 2008; Seale et al., 2008). It is therefore not surprising that a role for *Taf7l* in adipocytes could have been overlooked in previous studies. This cautionary tale also suggests that other key transcriptional regulators could likewise go undetected; suggesting that more studies will likely be required to take a fuller accounting of the multiple factor combinations that have evolved to orchestrate the diversity of gene regulatory pathways during differentiation and development of metazoans.

Although spermatogenesis and adipogenesis represent two biologically distinct differentiation processes, their common dependence on *Taf7l* may imply some underlying and perhaps hidden commonality. For example, one can speculate that energy storage and metabolic homeostasis are tightly related/linked to reproductive abilities since both are highly sensitive to and dependent on nutrient availability (Vernon et al., 1986; Hashmi et al., 2011). Another possible link involved steroid hormones (i.e. testosterone), which are essential for spermatogenesis, are derived from cholesterol (Chowdhury and Mukherjee, 1976; Mahmoud et al., 1985). In any case, our genome-wide mRNA-seq expression analysis unambiguously identified *Taf7l*-mediated enrichment of genes involved in both adipogenesis and reproductive processes. However, further investigation will be required to assess whether there are more direct links between adipogenesis and spermatogenesis and whether mutant *Taf7l* involved in male infertility also disturbs normal fat development and metabolism.

Our data clearly showed that TAF7L binds to and regulates the expression of a subset of white adipose genes, interestingly we also observed downregulation of some brown adipocyte genes (*Cidea*, *Ucp1* and *Elovl3*) caused by TAF7L knockdown (Figure 2C) (Kajimura et al., 2009; Kajimura et al., 2010). However, our incipient attempts failed to pinpoint significant defects in interscapular BAT formation in *Taf7l* KO mice. Thus, future studies will be required to determine whether *Taf7l* is also involved in BAT development. Further, our preliminary attempts to measure metabolic functions suggest that *Taf7l*-deficient mice showed changed serum glucose levels after 24 hr fasting compared with control WT littermates, it will be interesting to explore whether and how T*af7l* KO alters energy metabolism. Therefore, further detailed investigations will be required to more rigorously delineate the potential function of *Taf7l* in energy homeostasis.

## Materials and methods

### Vectors and plasmids

Mouse *Taf7l* full length cDNA was isolated from mouse testis tissue, amplified by PCR and then sequenced. Full length *Taf7l*, *Taf7*, *and Pparγ* cDNAs were inserted into pCMV 3×FLAG-10 vector to construct pCMV 3×FLAG-TAF7L/TAF7/PPARγ. *Taf7l*, *Tbp*, and *Pparγ* full length cDNAs were cloned into the pCS2+ vector with either HA or FLAG tag at their N-terminus. pLKO.1 shGFP and pLKO.1 shTAF7L vectors were purchased from Open Biosystems. shTAF7L-resistant pCMV 3×Flag-TAF7LmA was made by site-directed mutagenesis to introduce two silent mutations in shTAF7L targeted TAF7L cDNA sequence (Ding et al., 2008).

### Antibody production and purification, antibodies

A fragment of the *Taf7l* cDNA corresponding to residues 400–600 was cloned into the pGEX-4T-1 vector carrying a GST tag. GST-TAF7L (a. a. 400–600) was expressed and purified from *E.coli* and injected into rabbits by Covance (Covance Research Products Inc.,Denver, PA). Bleeds were collected after three boosts and TAF7L antibodies were tested and confirmed by in vitro transcribed and translated TAF7L protein and whole protein extracts from mouse testis of WT and *Taf7l* KO mice (data not shown). The antisera obtained were affinity-purified using antigen immobilized on Affigel 10/15 resin (Bio-rad, Hercules, CA). For Pol II antibody, monoclonal anti-Pol II (8WG16) was concentrated from hybridoma supernatant with Protein A Sepharose Beads (GE Healthcare, Piscataway, NJ).

Antibody information: anti-TAF4 (BD 612054), anti-TBP (abcam 62126), anti-FLAG (Sigma, F3165), anti-HA (abcam 9110), anti-β-actin (Sigma, A2228), anti-TAF7 (abnova H00006879-M01), anti-FABP4 (abcam 66682), Pol II (monoclonal 8GW16, protein-A purified), PPARγ (sc-7196), mouse and rabbit IgG (prepared in-house and concentrated with Protein A Sepharose Beads).

### Cells culture, stable cell line establishment

C3H10T1/2, 3T3-L1, C2C12, HeLa, and 293T cells were cultured in high glucose DMEM with 10% fetal bovine serum at 10% CO_2_.

C3H10T1/2 shGFP and shTAF7L lentiviral shRNA knockdown stable cell lines were established by transfecting pLKO.1 shGFP or pLKO.1 shTAF7L into C3H10T1/2 cells and then subjecting to puromycin selection for 3 weeks.

C3H10T1/2 shTAF7L cells were transfected with pCMV 3×Flag vector, pCMV 3×Flag-TAF7 or pCMV 3×Flag-TAF7LmA and then subjected to G418 and puromycin double selection for 3 weeks to establish shTAF7L+vector, shTAF7L+TAF7 or shTAF7L+TAF7LmA cell lines used in ‘rescue’ experiments in Figure 2E,F.

C2C12.CNTL and C2C12.TAF7L were established by transfecting pCMV 3×Flag vector or pCMV 3×Flag-TAF7L vector into C2C12 cells and then underwent G418 selection for 3 weeks.

### Adipocyte differentiation, Oil red O staining and C2C12 myogenesis

For adipogenesis, 3T3-L1 and C3H10T1/2 cells were grown in high glucose DMEM supplemented with 10% fetal bovine serum. At confluence, cells were exposed to induction medium containing dexamethasone (1 μM), isobutylmethylxanthine (IBMX, 0.1 mM), insulin (5 μg/ml), rosiglitazone (1 μM), and 10% FBS. 3 days later, cells were further cultured in high glucose DMEM containing insulin (5 μg/ml) and rosiglitazone (1 μM) until they were ready for harvest. C3H10T1/2 cells form mature adipocytes 5 days post induction; 3T3-L1 cells require 7–8 days to form adipocytes.

For Oil red O staining, pre- and post-differentiated C3H10T1/2 and 3T3-L1 cells, WT and *Taf7l* KO adipose-derived primary fibroblasts, shGFP and shTAF7L-treated C3H10T1/2 cells, C2C12.CNTL and C2C12.TAF7L cells were washed once in PBS and fixed with freshly prepared 4% formaldehyde in 1×PBS for 30 min, followed by standard Oil red O staining method described previously (Tang et al., 2004).

For C2C12 myogenesis, C2C12 cells are cultured in maintenance media until confluence was reached. 2 days post confluence, cells were switched to differentiation media comprised of low glucose DMEM, 2% horse serum, and 5 μg/ml insulin, 3 days later, change fresh differentiation media and culture cells for additional 2 days, differentiated myotubes were harvested and purified by collecting the suspended cells after splitting and reseeding the cells for 1 hr.

### RNA isolation and real-time PCR analysis

Total RNA from cultured cells or mouse tissues was isolated using QIAGEN RNeasy Plus mini columns according to the manufacturer's instructions (Qiagen Inc., Germantown, MD). For RT-qPCR analysis, 1 μg total RNA was reverse transcribed using cDNA reverse transcription kit (Invitrogen, Carlsbad,, CA). SYBR green reactions using the SYBR Green PCR Master Mix (Applied Biosystems, Warrington, UK) were performed according to the manufacturer's instruction using an ABI 7300 real time PCR machine (Applied Biosystems, Foster City, CA). Relative expression of mRNA was determined after normalization to total RNA amount. Student's t-test was used to evaluate statistical significance.

### Western blot analysis, immunoprecipitation

Whole cell extracts were prepared from cells by homogenization in lysis buffer containing 50 mM Tris–Cl, pH 8.0, 500 mM NaCl, and 0.1% Triton X-100, 10% glycerol and 1 mM EDTA, supplemented with protease inhibitor cocktail (Roche, Indianapolis, IN) and phenylmethylsulphonyl fluoride (PMSF). Fifteen micrograms (μg) of whole-cell lysates were separated by SDS-PAGE and transferred to nitrocellulose membrane. For immunoblotting, membranes were blocked in 10% milk, 0.1% Tween-20 in TBS for 30 min, and then incubated with TAF7L, TAF4, TAF7, FLAG, β-actin, PPARγ and TBP antibodies for 2 hr at room temperature; detailed Western blotting procedure was performed as previously described (Zhou et al., 2006).

500 μg whole-cell extracts from 293T cells transfected with FLAG-TAF7L and HA-PPARγ, FLAG-PPARγ and HA-TBP, or FLAG-TAF7L and HA-TBP were immunoprecipitated with FLAG or HA antibodies at 4°C for overnight under the conditions of 0.3 M NaCl and 0.2% NP-40, 30 μl protein A/G beads were added and incubated for additional 2 hr at 4°C, after extensive washing with buffer containing 0.15 M NaCl and 0.1% NP-40, remaining beads were subjected to 10% SDS-PAGE and followed by western blotting analysis with FLAG and HA antibodies to detect tagged-proteins in the inputs and IPs as previously described (Ding et al., 2008).

### Animals and genotype analysis

The derivation of *Taf7l*-knockout mice has been previously described (Cheng et al., 2007). All animal experiments were performed in strict accordance with the recommendations in the Guide for the Care and Use of Laboratory Animals of the National Institutes of Health. All of the animals were handled according to approved animal use protocols (#R007) by Animal Care and Use Committee (ACUC) of the University of California, Berkeley. Mice were maintained on a standard rodent chow diet with 12 hr light and dark cycles. *Taf7l* KO mouse line was maintained on a C57/Bl6 background. Genotyping was performed by PCR as previously described (Cheng et al., 2007).

### Preparation of primary fibroblast and induction of adipogenesis

Fresh inguinal adipose tissues were removed from 3 week old euthanized WT and *Taf7l* KO mice and finely minced, digested with 0.25% trypsin for 30 min at 37°C, and centrifuged for 5 min at 2,000*g*. The pellet was resuspended in culture media before plated on gelatin coated plates. Cells were cultured at 37°C in high glucose DMEM supplemented with 20% FBS. Adipocyte differentiation and staining were followed the same procedure as C3H10T1/2 cells.

### Immunohistochemistry

For histological analysis on interscapular tissue of E18.5 embryos from WT and *Taf7l* KO mice, freshly-harvested mouse embryos were genotyped and then interscapular regions of embryos were transversally dissected and then fixed in 10% formaldehyde for 24 hr at 4°C; tissue was embedded in paraffin using the microwave method and then sectioned into 8–10 μm sections to mount on slides. This method and the following immunohistochemistry by haematoxylin and eosin (H&E) staining were performed using the method described previously by Steven Ruzin (Schichnes et al., 1998), and FABP4 immunostaining method was modified from the one described previously (Kajimura et al., 2008) (Gupta et al., 2010).

### ChIP, ChIP libraries preparation, and deep sequencing (ChIP-seq)

Fix C3H10T1/2 cells and differentiated adipocytes with 1% formaldehyde for 10 min at room temperature then use 0.125 M glycine to stop the crosslinking for an additional 5 min. Collect cells and extract nuclei with extraction buffer. The chromatin obtained from C3H10T1/2 cells and adipocytes was fragmented to sizes ranging from 175 to 225 bp using a Covaris-S2 sonicator (Covaris, Inc., Woburn, MA) for a total processing time of 40 min (20 s on, 20 s off). 900 µg of the sonicated chromatin was used in each immunoprecipitation reaction as previously described (Liu et al., 2011) with the Pol II, TBP, PPARγ, and TAF7L antibodies, mouse and rabbit IgG were used as negative controls respectively. Preparation of the sequencing libraries on the DNA samples of the immunoprecipitation from antibodies and IgGs precisely followed the instructions from Illumina (Illumina Inc., San Diego, CA), qualities of the libraries were assessed by 2100 Bioanalyzer (Functional Genomics Laboratory, Berkeley,CA) and then subjected to ultra-high throughput sequencing on an Illumina HiSeq 2000 sequencer (GSL core facility, Berkeley, CA) as previously described (Fong et al., 2011; Liu et al., 2011), each sample yielded ∼50–200 million reads.

### Mapping sequencing reads to the genome

Sequenced reads were mapped to the July 2007 assembly of the mouse genome (UCSC version mm9, NCBI37) using Bowtie (Langmead et al., 2009) with the command-line options ‘-c -q -n 2 -l 48 -m 1’, thereby keeping for further analyses only reads that mapped uniquely to the genome with at most two mismatches at the first 48 bases.

### Peak calling methods

To accurately identify significant binding positions across the genome, we incorporated two peak calling methods, as described previously (May et al., 2012). First, we used MACS (Zhang et al., 2008) (version 1.4, with default settings except: --nomodel --shiftsize = 110 --pvalue=1e-2 --mfold = 10,10,000 --slocal = 2000 --llocal = 20,000), with an FDR significant threshold of 0.05 (using IgG ChIP as a control). Second, we applied the Grizzly peak fitting algorithm (Harrison et al., 2011), which uses a model-based iterative approach to accurately identify multiple binding loci at every enriched region. Peaks were then associated with the nearest TSS (using gene annotation from UCSC, version mm9), and classified as promoter peaks (up to 500 bp from start site), proximal enhancer peaks (larger than 500 bp and less than 5 kb), distal enhancer peaks (less than 50 kb from TSS), or none (further away than any start site).

### Motif identification

The DNA sequence associated with each of the peaks is a 250 bp fragment that is centered on the highest point peak within the TAF7L binding region as defined by Grizzly Peak. Weeder (version 1.4.2) (Pavesi et al., 2004) searches the inputted sequences for enriched motifs. The Weeder output is then passed through STAMP (Mahony and Benos, 2007) to compare the identified motif's frequency matrix against the JASPAR v2010 database and identify which transcription factors have that DNA binding motif.

### mRNA-seq libraries preparation and deep sequencing

Total RNAs were extracted from C3H10T1/2 cells and adipocytes, differentiated shTAF7L-C3H10T1/2 cells, differentiated C2C12.CNTL and C2C12.TAF7L cells by RNeasy Plus Mini Kit (Qiagen), 8 μg of each sample was used to purify mRNA and subsequently converted into to mRNA-seq library using mRNA-Seq Sample Prep Kit (Illumina) and sequenced on an Illumina HiSeq 2000 sequencer. 50 bp paired-end were used for the C3H10T1/2 samples and 100 bp single-end were used for the C2C12 samples, both resulting in over 175 million reads yield per sample.

### Digital gene expression of mRNA-seq

The reads were than mapped to the mouse transcriptome (created using UCSC table browser, version mm9, on February 2012), using TopHat (Trapnell et al., 2009), version v1.4.0., using default parameters. We then applied cufflinks (Trapnell et al., 2010), version v1.3.0, using the default parameters except: --max-mle-iterations 1, to estimate the digital expression levels at each transcript. Due to the high number of sequence reads, these steps were done in seven batches of 25 million reads each, per sample.

### Data availability

Raw and mapped sequencing reads are available from the National Center for Biotechnology Information's GEO database (http://www.ncbi.nlm.nih.gov/geo/) under accession number GSE41937. All supplemental data, including gene-by-gene ChIP-seq and gene expression mRNA-seq data, are available at http://eisenlab.org/data/TAF7L. A genome browser with mapped mRNA and ChIP profiles and other related data discussed in the manuscript can be accessed at http://eisenlab.org/data/TAF7L/browse or directly at http://genome.ucsc.edu/cgi-bin/hgTracks?hgS_otherUserName=tomkap&hgS_otherUserSessionName=TAF7L

## Additional files

### Major datasets

The following datasets were generated:

ZhouH, KaplanT, LiY, GrubisicI, ZhangZ, WangPJ, EisenMB, TjianR, 2012, Dual Functions of TAF7L in Adipocyte Differentiation, ; http://www.ncbi.nlm.nih.gov/geo/query/acc.cgi?acc=GSE41937; http://eisenlab.org/data/TAF7L, In the public domain at GEO: http://www.ncbi.nlm.nih.gov/geo/

The following previously published datasets were used:

MikkelsenTS, XuZ, ZhangX, WangL, GimbleJM, LanderES, RosenED, 2010, Epigenomic profiling of 3T3-L1 adipogenesis, ; http://www.ncbi.nlm.nih.gov/geo/query/acc.cgi?acc=GSE21365, In the public domain at GEO: http://www.ncbi.nlm.nih.gov/geo/

SchmidtSF, JorgensenM, ChenY, NielsenR, SandelinA, MandrupS, 2011, Cross-species comparison of C/EBPα and PPARγ profiles in mouse and human adipocytes reveals interdependent retention of binding sites, ; http://www.ncbi.nlm.nih.gov/geo/query/acc.cgi?acc=GSE27450, In the public domain at GEO: http://www.ncbi.nlm.nih.gov/geo/

WakiH, NakamuraM, YamauchiT, WakabayashiK, YuJ, Hirose-YotsuyaL, TakeK, SunW, IwabuM, Okada-IwabuM, FujitaT, AoyamaT, TsutsumiS, UekiK, KodamaT, SakaiJ, AburataniH, KadowakiT, 2011, Genome-wide profiling of PPARg and RXRa and CTCF and H3K4me3 in Adipocytes, ; http://trace.ddbj.nig.ac.jp/DRASearch/study?acc=DRP000383, In the public domain at the DNA Data Bank of Japan: http://www.ddbj.nig.ac.jp/
